# Activation-induced cytidine deaminase prevents pro-B cell acute lymphoblastic leukemia by functioning as a negative regulator in Rag1 deficient pro-B cells

**DOI:** 10.18632/oncotarget.20563

**Published:** 2017-09-07

**Authors:** Franziska Auer, Deborah Ingenhag, Stefan Pinkert, Sven Kracker, Salima Hacein-Bey-Abina, Marina Cavazzana, Michael Gombert, Alberto Martin-Lorenzo, Min-Hui Lin, Carolina Vicente-Dueñas, Isidro Sánchez-García, Arndt Borkhardt, Julia Hauer

**Affiliations:** ^1^ Department of Pediatric Oncology, Hematology and Clinical Immunology, Heinrich-Heine University Duesseldorf, Medical Faculty, Duesseldorf, Germany; ^2^ Université Paris Descartes, Sorbonne Paris Cité, Imagine Institute, Paris, France; ^3^ INSERM UMR 1163, Human Lymphohematopoiesis Laboratory, Paris, France; ^4^ UTCBS CNRS UMR 8258, INSERM U1022, Faculté de Pharmacie de Paris, Université Paris Descartes, Sorbonne Paris Cité, Chimie Paris-Tech, Paris, France; ^5^ Clinical Immunology Laboratory, Groupe Hospitalier Universitaire Paris-Sud, Hôpital Kremlin-Bicêtre, Assistance Publique-Hôpitaux de Paris, Le-Kremlin-Bicêtre, France; ^6^ Experimental Therapeutics and Translational Oncology Program, Instituto de Biología Molecular y Celular del Cáncer, CSIC/ Universidad de Salamanca, Campus M. de Unamuno s/n, Salamanca, Spain; ^7^ Institute of Biomedical Research of Salamanca, Salamanca, Spain

**Keywords:** acute lymphoblastic leukemia, activation induced cytidine deaminase, pro-B cells, Rag1 deficiency

## Abstract

Activation-induced cytidine deaminase (AID) is essential for somatic hypermutation and class switch recombination in mature B-cells, while AID was also shown to play a role in developing pre-BCR/BCR-positive B-cells of the bone marrow. To further elucidate a potential function of Aid in the bone marrow prior to V(D)J-recombination, we utilized an *in vivo* model which exerts a B-cell developmental arrest at the pro-B cell stage with low frequencies of pro-B cell acute lymphoblastic leukemia (pro-B ALL) development. Therefore, *p19Arf*^-/-^*Rag1*^-/-^ (AR) mice were crossed with Aid-deficient mice (ARA). Surprisingly, loss of Aid expression in pro-B cells accelerated pro-B ALL incidence from 30% (AR) to 98% (ARA). This effect was Aid dose dependent, since *Aid*^+/-^ animals of the same background displayed a significantly lower incidence (83%). Furthermore, B-cell-specific Aid up-regulation was observed in Aid-competent pro-B ALLs. Additional whole exome/sanger sequencing of murine pro-B ALLs revealed an accumulation of recurrent somatic Jak3 (p.R653H, p.V670A) and Dnm2 (p.G397R) mutations, which highlights the importance of active IL7R signaling in the pro-B ALL blast cells. These findings were further supported by an enhanced proliferative potential of ARA pro-B cells compared to Aid-competent cells from the same genetic background. In summary, we show that both Aid and Rag1 act as a negative regulators in pro-B cells, preventing pro-B ALL.

## INTRODUCTION

Over the last decade, various studies gave novel insights that improved our understanding about the molecular basis of childhood acute lymphoblastic leukemia (ALL) [1–5]. Genomic alterations are implicated in disease progression of B-cell precursor ALL, and *RAG1* is the key player in this context [6–11]. Unexpectedly, RAG1 loss of function mutations were detected in bone marrow (BM) samples of ALL patients despite the presence of several genomic copy number alterations [12], which suggests that molecules other than RAG1 are able to induce genomic alterations in the respective blast cells. In addition to the recombination-activating genes RAG1 and RAG2, which are responsible for V(D)J-recombination [13], the activation-induced cytidine deaminase (AID) enzyme is necessary to produce the secondary repertoire of antibodies in germinal center B-cells [14]. By deaminating cytosine residues in Ig variable as well as switch regions, AID is responsible for somatic hypermutation (SHM) and class-switch recombination (CSR) [15, 16].

While AID expression was shown to have implications in B-cell lymphomas [17, 18], which resemble a mature B-cells stage, aberrant AID activity could furthermore be linked to BCR-ABL positive leukemia [19], a disease affecting B-cell precursors. Although the role of AID has been elaborately studied in the context of germinal center B-cells, recent evidence highlighted how AID already exerts a functional role in developing B-cells [8, 20]. While it was shown that the concurrent expression of AID and RAG1 in small pre-BII cells contributes to the clonal evolution of childhood ALL in the presence of strong inflammatory stimuli [8], absence of AID expression in pre-BI and immature B-cells has been reported to confer implications in the control of self-tolerance, as shown in both mice and humans [20–25]. Up to now, functional AID expression in the BM could be detected in small pre-BII [8], early immature [26] and transitional-1 B-cells [27, 28]. Whether AID is already utilized by earlier B-cell precursors that do not express a precursor B-cell receptor (pre-BCR) is still controversially discussed. In order to elucidate whether Aid is indeed functional prior to pre-BCR expression, we developed an Aid-deficient mouse model with a tumor prone *Rag1*^-/-^ background (*p19Arf*^-/-^*Rag1*^-/-^*Aid*^-/-^ ARA). Utilizing this model, we were able to assess the influence of Aid at the pro-B cell stage [29], in the context of pro-B ALL development [30] and in the absence of Rag1 induced alterations. Here, we present *in vivo* evidence, that the combined absence of Aid and Rag1 in tumor prone murine pro-B cells accelerates pro-B ALL incidence, which suggests a functional role of Aid in Rag1 deficient BM pro-B-cells even before the expression of a pre-BCR.

## RESULTS

### Aid is a negative regulator of pro-B ALL development in *Rag1*^-/-^ tumor-prone mice

**Figure 1:**
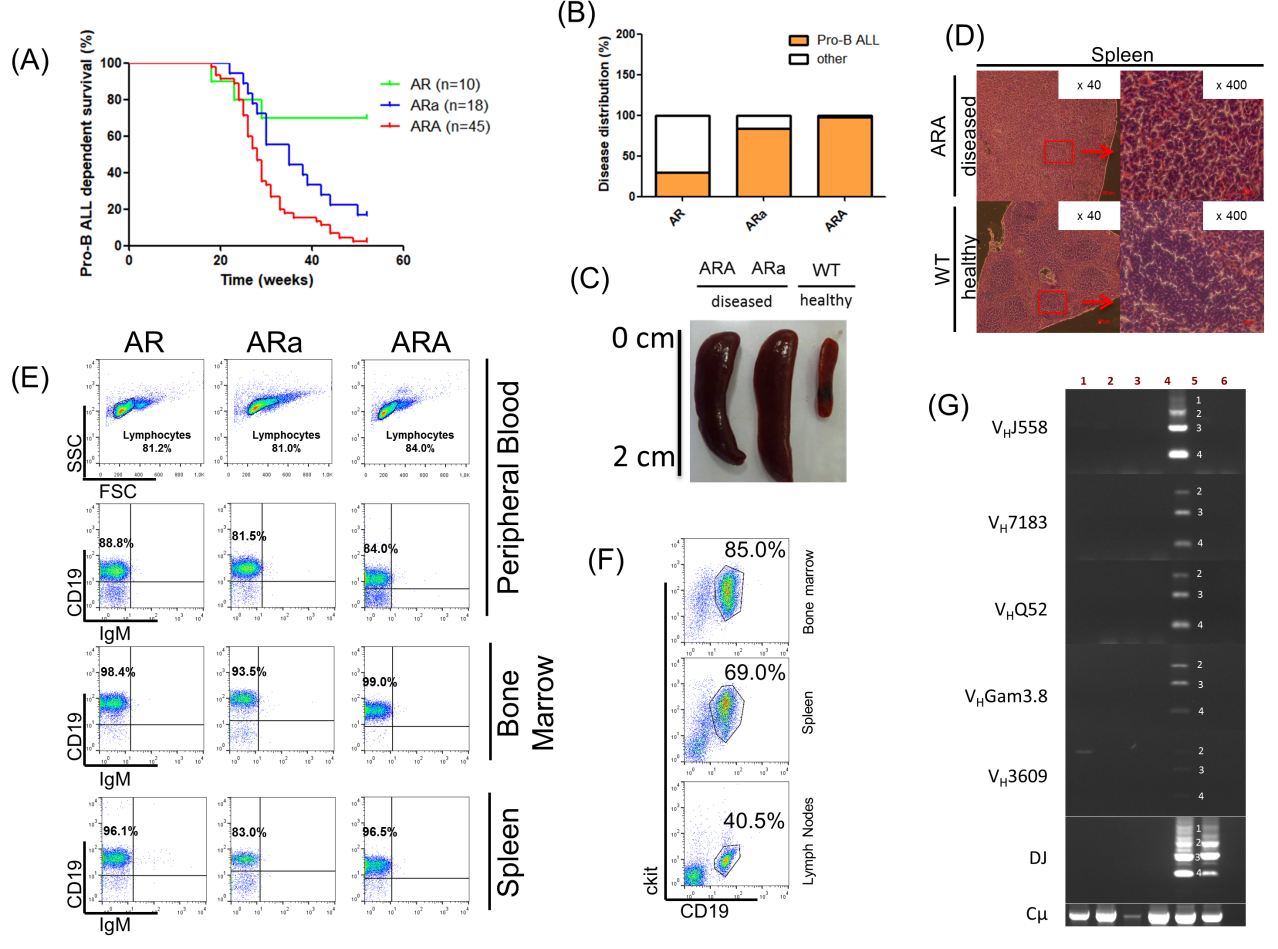
Aid deficiency accelerates pro-B ALL development in *p19Arf*^-/-^*Rag1*^-/-^ mice **A.** Pro-B-ALL-dependent survival curve of *p19Arf*^-/-^*Rag1*^-/-^ (AR), *p19Arf*^-/-^*Rag1*^-/-^*Aid*^+/-^ (ARa) and *p19Arf*^-/-^*Rag1*^-/-^*Aid*^-/-^ (ARA) mice. Curves show a significant difference in pro-B-ALL-dependent survival (Log-rank test *p* < 0.0001). **B.** Disease distribution of pro-B ALL in AR, ARa and ARA mice. **C.** Representative splenomegaly of a diseased ARa and ARA mouse, compared to a C57BL/6J wildtype mouse. **D.** Hematoxylin/Eosin staining from leukemic ARA spleens, showing loss of their architecture due to blast cell infiltration. **E.** Representative blot of hematopoietic subsets in diseased AR, ARa and ARA mice, showing an accumulation of CD19^+^IgM^-^ pro-B cells. **F.** Representative FACS analysis of a non-irradiated C57BL/6J wildtype recipient mouse that was transplanted with leukemic total BM from a diseased ARA donor mouse. Nine days after BM transplantation, blast cells (CD19^+^ckit^+^) are visible in bone narrow, spleen and lymph nodes (*n* = 2). **G.** Immunoglobulin V(D)J-recombination in ARa (lanes 1,2) and ARA (lanes 3,4) tissues infiltrated with leukemic blast cells, as analyzed by PCR. Thymocytes (lane 6) serve as negative control and sorted CD19^+^ B-cells (lane 5) from the spleens of healthy C57BL/6 wildtype mice serve as a control for polyclonal V(D)J-recombination. Infiltrated tissues show only the cμ heavy chain.

In order to explore the physiological relevance of Aid at the pro-B cell stage, and independent of Rag1 off-target activity, we designed a mouse model which allows the investigation of an arrested tumor-prone pro-B cell population in combination with Aid deficiency. Aid knockout mice (Aid-/-) were crossed with p19Arf-/-Rag1-/- (AR) mice to obtain p19Arf-/-Rag1-/-Aid-/- (ARA) and p19Arf-/-Rag1-/-Aid+/- (ARa) mice. P19Arf-/-Rag1-/- mice are known to develop pro-B ALL at a rate of 26% [30], which we were able to reproduce in our own independent cohort that showed a pro-B ALL incidence of 30% (Figure 1A). Surprisingly, concomitant Aid deficiency accelerated the pro-B ALL incidence to 98% (Log-rank test p < 0.001). Moreover, this effect was shown to be Aid dose dependent, since the Aid heterozygous (ARa) animals of the same background displayed significantly lower pro-B ALL development (83%, Log-rank test p = 0.0167) (Figure 1A). Other disease phenotypes observed in ARa animals included solid tumors (5.56%) and myeloproliferative diseases (11.11%), while AR animals predominantly developed solid tumors in contrast to the pro-B ALLs in ARA mice [30] (Figure 1B). The leukemias manifested with splenomegaly (Figure 1C and Supplementary Figure 4A), disrupted splenic architecture related to blast cell infiltration (Figure 1D), and appeared in the peripheral blood (PB) (Figure 1E). FACS analysis revealed a pro-B cell surface phenotype CD19+ckit+IgM- for blast cells, which extended through BM, PB and spleen (Supplementary Figure 1-3). Moreover, pro-B ALLs were able to engraft in secondary recipients with a phenotype identical to the primary disease and a latency of 1-2 weeks (Figure 1F). V(D)J-recombination assays verifying only the presence of the cμ chain were performed to ensure that the model was not leaky, and that pro-B ALL was not derived from mature AID-expressing B-cells which had undergone V(D)J-recombination (Figure 1G and Supplementary Figure 4B). Hence, the leukemia arose from pro-B cells, which were unable to undergo Rag1-mediated V(D)J-recombination. These results suggest a dose-dependent and protective Aid function in Rag1-/- pro-B cells even before the development of a functional pre-BCR.

### Reduced Aid expression correlates with pro-B ALL incidence in *Rag1*^-/-^ tumor-prone mice

**Figure 2:**
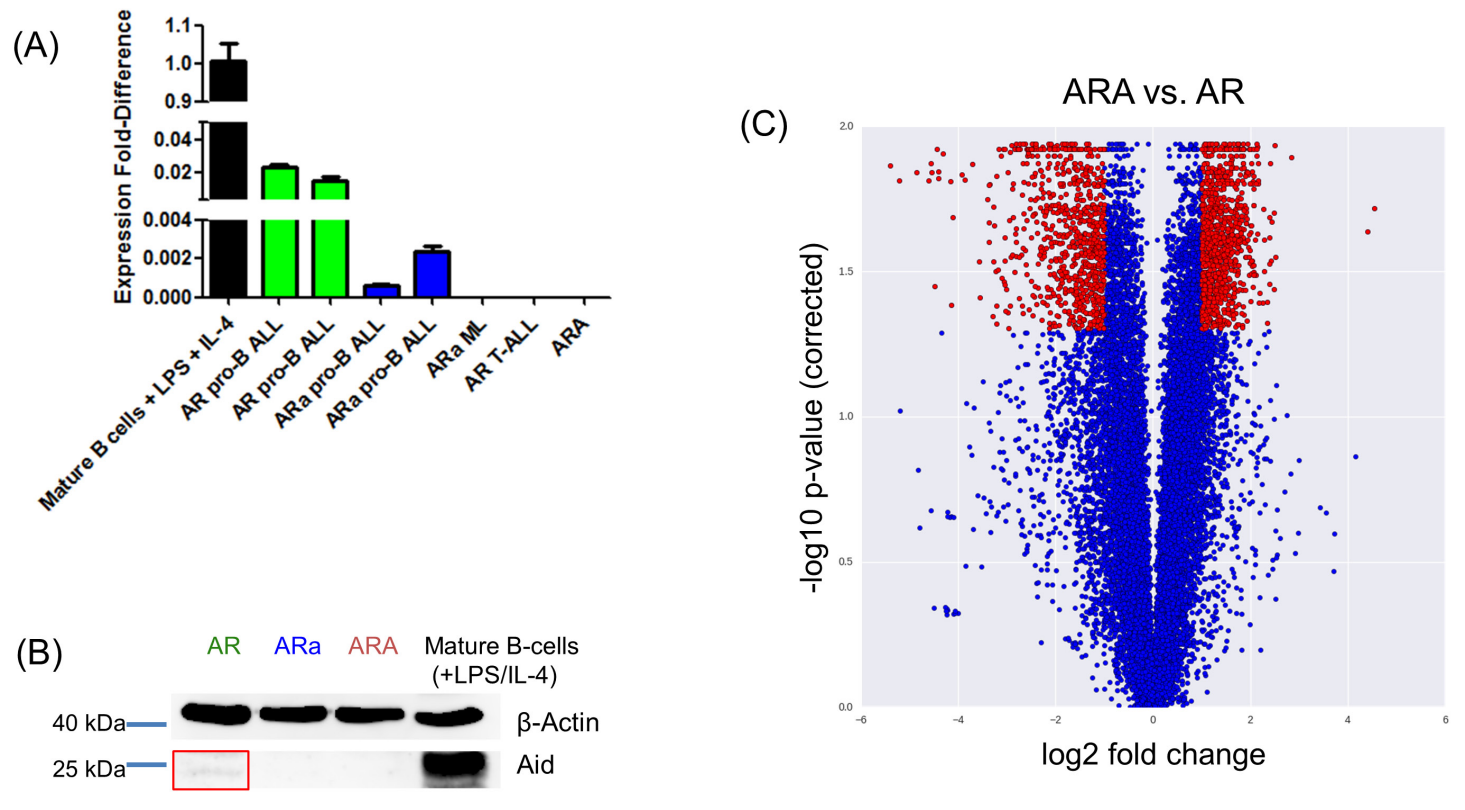
B-cell-specific Aid expression detectable in *p19Arf*^-/-^*Rag1*^-/-^ mice **A.** Quantitative Real-time-PCR analysis showing Aid mRNA expression levels in *p19Arf*^-/-^*Rag1*^-/-^ (AR), *p19Arf*^-/-^*Rag1*^-/-^*Aid*^+/-^ (ARa) and *p19Arf*^-/-^*Rag1*^-/-^*Aid*^-/-^ (ARA) tumors. Stimulated B220^+^ splenic B-cells (LPS/IL-4) from wildtype mice serve as positive control. Aid expression is detectable in AR and ARa pro-B ALLs, while it is absent in the respective T-ALLs and myeloproliferative diseases (ML) (*n* = 3). **B.** Immunoblot analysis showing the presence of the Aid protein in AR tumors. Leukemic blast cells of ARA mice serve as a negative control, while stimulated B220^+^ splenic B-cells from wildtype mice were used as a positive control. Beta-Actin serves as a loading control (*n* = 3). **C.** Microarray analysis results comparing the gene expression between AR and ARA tumor samples, which were visualized in a volcano plot. Red dots have a fold change greater than two and a corrected p-value smaller than 0.05. Out of the 18465 data points, 823 are lower and 1001 higher in ARA tumor samples relative to AR tumors.

Thus far, it has been proposed that physiologic AID expression in the BM is restricted to CD19^+^ B-cells, which co-express a functional IgM heavy chain product [22], since AID transcripts are first detected in pre-BII and immature B-cells, but not in earlier B-cell precursor populations [8]. Here, we show that Aid is expressed in tumors from *p19Arf*^-/-^*Rag1*^-/-^ (AR) mice, which were classified as Hardy Fractions B, resembling a pro-B stage [30]. While no Aid expression was detected in sorted B220^+^ BM cells from healthy AR mice (data not shown), significant Aid expression was observed in AR pro-B ALLs (Figure 2A). The presence of Aid was further confirmed on protein level using immunoblotting (Figure 2B and Supplementary Figure 4D). Moreover, the detected Aid expression was shown to be dose dependent, since in ARa pro-B ALLs Aid expression was 10-30 times lower compared to AR leukemias (Figure 2A). In AR and ARa mice, a small proportion of T-ALL and myeloid neoplasia occurred with no detectable Aid expression and, therefore, we consider Aid expression to be B-cell specific (Figure 2A and Supplementary Figure 4C). Further microarray analysis comparing ARA to AR pro-B ALLs validated a differentially regulated gene expression profile between both groups (1774 genes significantly up and 1566 genes significantly down) (Figure 2C and Supplementary Table 1). Since the reduction and loss of Aid expression at the Rag1 deficient pro-B cell stage accelerated the leukemia incidence, we suggest Aid expression as a negative regulator in Rag1 deficient pro-B cells to clear pre-leukemic populations.

### Murine tumor profiling reveals somatic mutations influencing the IL7R/Jak3/Stat5-axis

**Figure 3:**
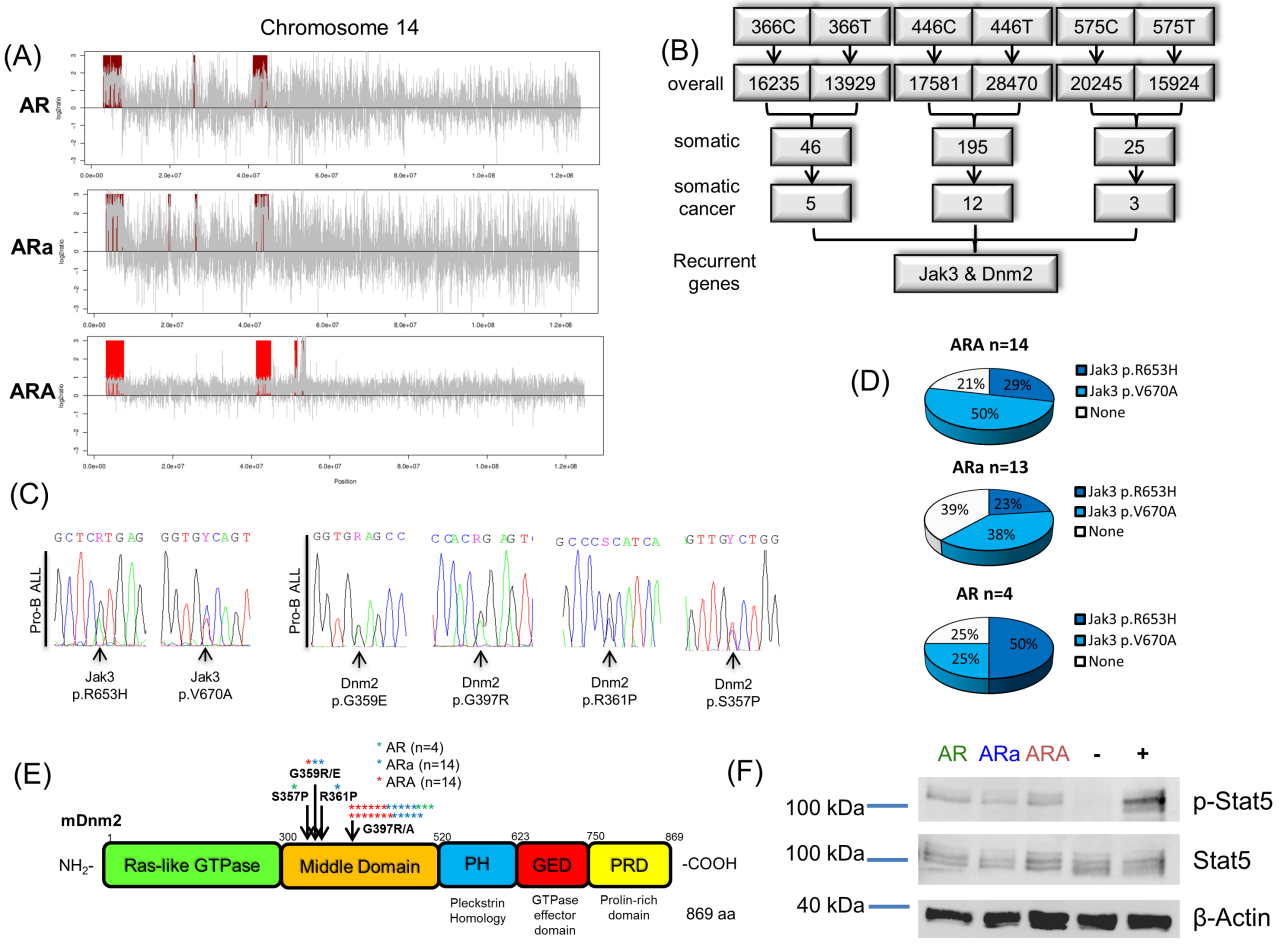
Sequencing of Aid-deficient tumors reveals activating mutations influencing IL-7 signaling **A.** From Tumor/reference pairs of leukemic *p19Arf*^-/-^*Rag1*^-/-^ (AR), *p19Arf*^-/-^*Rag1*^-/-^*Aid*^+/-^ (ARa) and *p19Arf*^-/-^*Rag1*^-/-^*Aid*^-/-^ (ARA) mice, CNV profiles were calculated using EXCAVATOR2. A representative plot for each genotype is shown. CNV profiles display recurrent gains (red) of regions on chromosome 14. **B.** Presentation of WES analysis from three ARA pro-B ALLs. 5-11 of the somatic variants were deemed tumor-specific by MUTECT analysis, with *Jak3* and *Dnm2* as recurrent genes. **C.** Representative chromatogram of Jak3 (p.R653H/p.V670A) and Dnm2 (p.G359E/p.G397R/p.R361P/p.S357P) mutation validation by Sanger sequencing. **D.** Sanger sequencing results of *Jak3* Exon15 show an accumulation of somatic Jak3 p.R653H and p.V670A variants in AR (*n* = 4), ARa (*n* = 13) and ARA (*n* = 14) tumors. **E.** Sanger sequencing identifies recurrent mutations in the Dnm2 gene affecting the amino acids p.G359R/E, p.R361P, p.S357P and p.G397R/A in AR (*n* = 4), ARa and ARA pro-B ALLs (*n* = 14). **F.** Immunoblot analysis showing Stat5 phosphorylation in AR, ARa and ARA pro-B ALLs. IL-3 depleted BaF3 cells serve as negative (-) and BaF3 cells overexpressing murine Jak3 V670A as positive (+) control. Stat5 and beta-Actin were used as loading controls (*n* = 3).

To elucidate structural aberrations of the pro-B leukemias, copy number variation analysis of three ARA, three ARa and one AR tumor sample was carried out. Thereby, Chr. 14 amplifications were recurrently detected in all analyzed pro-B ALLs (Figure 3A, Supplementary Figure 5 and Supplementary Table 2). Although no cancer related genes could be found in the recurrently amplified regions, the analysis validates the integrity of the murine model, since the same aberrations were already described for AR leukemias [30]. Furthermore, the identified cytogenetic abnormality can be contributed to the p19Arf deficiency, since recurrent whole Chr. 14 gains were previously reported in ARFnull lymphomas of Eμ-myc transgenic mice and linked to an improved treatment response [31]. To further identify secondary somatically acquired hits, which lead to pro-B ALL development, we performed whole exome sequencing (WES) of three leukemic ARA BM tumor samples, and their corresponding germline DNA, which was extracted from the tail of the respective mouse at first signs of disease. Somatic variants identified by WES ranged from 26 to 195 per mouse, and of these 3 to 12 were deemed tumor specific according to the COSMIC cancer gene consensus list [32]. All three analyzed ARA pro-B ALLs displayed recurrent heterozygous variants in the Jak3 (p.R653H/p.V670A) and Dnm2 gene (p.G397R) (Figure 3B/3C). Sanger sequencing of the hotspot regions in both target genes in an extended cohort of AR, ARa and ARA pro-B ALLs, revealed a mutational pattern of somatic Jak3 mutations in 75% of AR, 61% of ARa and 79% of ARA mice (Figure 3D). Moreover, Dnm2 was somatically mutated in close to 100% of all pro-B ALLs analyzed, with all identified variants located in the middle domain (Figure 3E). Immunoblotting of leukemic AR, ARa and ARA BM samples validated active Stat5 signaling in all three cohorts (Figure 3F), which is in line with the detected activating Jak3 variants [33]. Moreover, it was recently reported for a Lmo2 transgenic mouse model that loss of function mutations in Dnm2 increase the IL-7R cell surface expression, which expands the pool of IL-7 responsive cells [34]. These findings highlight the importance of the IL-7 signaling pathway in pro-B leukemias.

### Aid deficiency correlates with enhanced proliferation of healthy pro-B cells in *Rag1*^-/-^ tumor-prone mice

**Figure 4:**
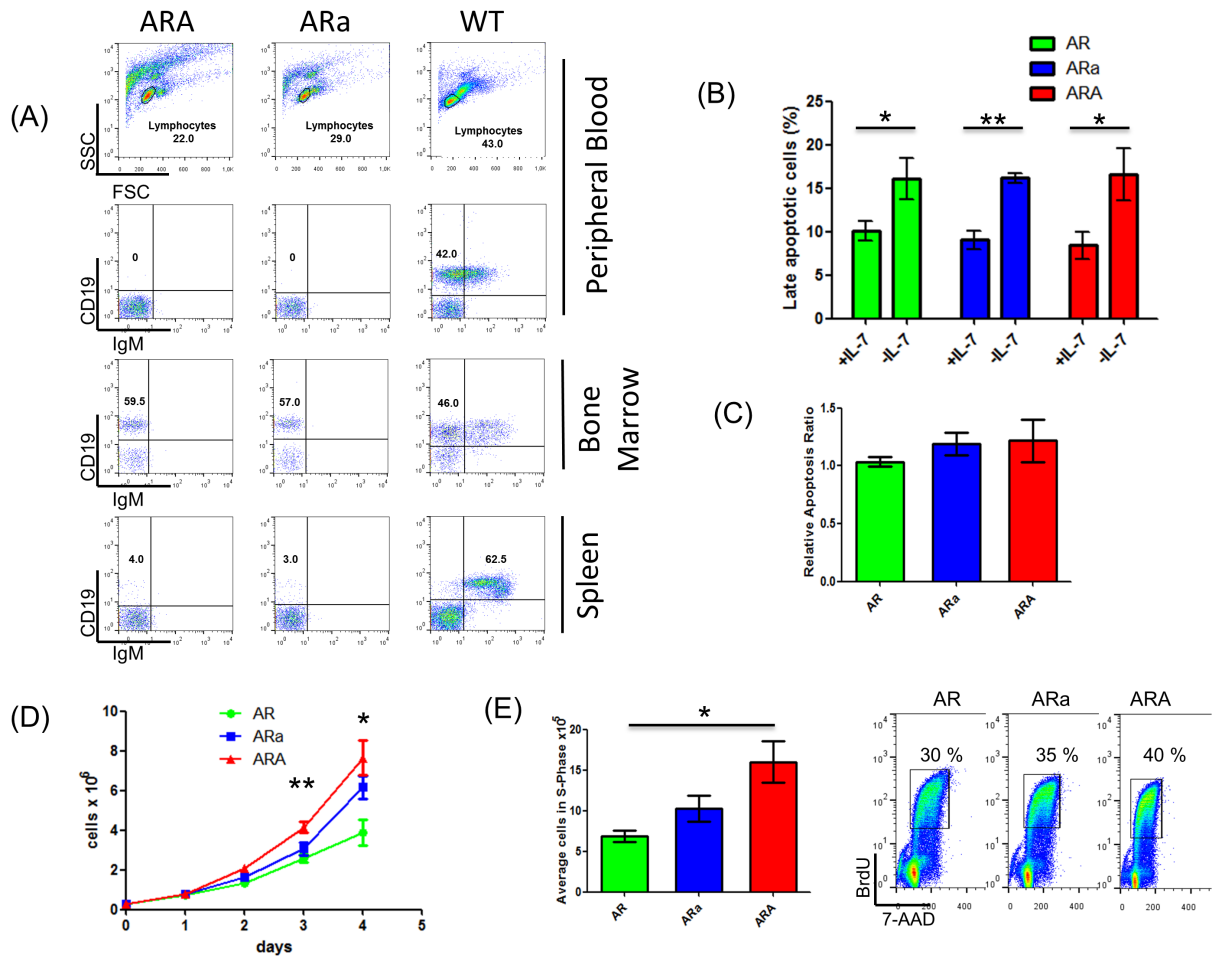
*In vitro* cultured pro-B cells of healthy *p19Arf*^-/-^*Rag1*^-/-^*Aid*^-/-^ mice are highly IL-7 dependent and show an increased proliferative potential compared to their Aid-competent counterparts **A.** Representative flow cytometric analysis of hematopoietic subsets in healthy *p19Arf*^-/-^*Rag1*^-/-^*Aid*^+/-^ (ARa) and *p19Arf*^-/-^*Rag1*^-/-^*Aid*^-/-^ (ARA) mice compared to wildtype mice. **B.** FACS analysis depicting the percentage of cells at a late apoptotic stage (7-AAD^+^Annexin^+^) from healthy *p19Arf*^-/-^*Rag1*^-/-^ (AR), ARa and ARA pro-B cells cultured *in vitro* after 24 hours of IL-7 withdrawal. All groups show significant late apoptosis induction as calculated by student's t-test (AR *p* = 0.0444; ARa *p* = 0.0049; ARA *p* = 0.05; *n* = 3 for AR/ARa; *n* = 4 for ARA). **C.** FACS analysis showing the relative apoptosis ratio of healthy AR, ARa and ARA pro-B cells after 24 hours of IL-7 depletion. Therefore, the ratio between 7-AAD/Annexin double positive cells between the groups in IL-7+ and IL-7- conditions was calculated and the AR ratio set to 1. No significant differences in IL-7 sensitivity were observed between the groups (*n* = 3 for AR/ARA; *n* = 4 for ARA). **D.** Proliferation curve of healthy AR, ARa and ARA pro-B cells. Compared to AR cells, ARA cells show significantly increased proliferation on day 3 (*p* = 0.0098) and day 4 (*p* = 0.0244) as calculated by student's t-test. **E.** Representative FACS analysis and blot showing cell cycle distribution of healthy AR, ARa and ARA pro-B cells after 1 hour of BrdU pulse labeling. ARA pro-B cells display increased accumulation of cells in the S-phase compared to AR pro-B cells (student's t-test, *p* = 0.0318; *n* = 3 for AR/ ARA; *n* = 4 for ARA).

Compared to WT mice, ARA and ARa mice showed an accumulation of CD19+ IgM- pro-B cells in the bone marrow (Figure 4A). To analyze whether Aid deficiency influences the pro-B cell population in young and healthy AR, ARa and ARA mice, B220^+^ BM B-cells from the respective backgrounds were sorted and cultured *in vitro*. Since the importance of the IL-7 signaling was highlighted in the somatic mutational spectrum, IL-7 sensitivity assays were performed. Thereby BM pro-B cells of healthy AR, ARa and ARA mice showed significant apoptosis induction (Figure 4B) (AR *p* = 0.0444, Ara *p* = 0.0049, ARA *p* = 0.05; student's t-test) while differences in IL-7 sensitivity could not be observed between the groups after 24 hours of IL-7 withdrawal (Figure 4C). These results emphasize the strong IL-7 dependency of the Rag1 negative pro-B cells, which interfered with the *in vitro* simulation of a potential Aid induction in the absence of IL-7 [8]. In this regard, *Aicda* transcripts were not detectable at 24 or 48 hours after IL-7 depletion in either AR or ARa healthy pro-B cells, even in the presence of LPS as infectious stimulus (data not shown). Nevertheless, the proliferation rate of healthy ARA B220^+^ B-cells was significantly higher (day 3 *p* = 0.0098, day4 *p* = 0.0244) compared to AR B220^+^ B-cells (Figure 4D). This observation extends the findings of Kuraoka *et al*. [21], stating that *Aicda*^-/-^ progenitors exhibit a significant advantage in the production of immature and mature B-cell compartments. This proliferative increase was further validated by BrdU assays, showing enhanced accumulation of ARA pro-B cells (*p* = 0.0318, student's t-test; AR = 26.8±4.8%, ARa = 33.4±9.2%, ARA = 38.5±12.2%) in the DNA synthesis phase (S-phase) (Figure 4E and Supplementary Figure 4E). Since the analyzed pro-B cells are highly IL-7 dependent, we were unable to investigate the observed results with regard to Aid induction in the absence of IL-7.

## DISCUSSION

In the BM, AID induction through inflammatory stimuli drives the clonal evolution of B-ALL [8], while its loss/reduction in immature B-cells [26] interferes with the negative selection at central tolerance checkpoints for the removal of autoreactive B-cells [20–22, 24, 35]. Here, we show that in a mouse model designed for the investigation of arrested pro-B cells, an additional loss of Aid accelerated the pro-B ALL incidence from 30% to 98%. This effect was dose dependent, since Aid heterozygous mice on the same background displayed significant disease reduction, which is in line with a previous report on Aid haploinsufficiency [36]. Moreover, these findings are in agreement with results from Cantaert *et al*., showing that AID gene dosage regulates central B-cell tolerance in humans [26]. AID-mediated CSR can be actively induced in B-cell precursors *in vivo* and *in vitro* [27, 37–39], which further suggests that the function of AID is also preserved at the pro-B cell stage. The fact that the Aid-competent mice in our model were more protected against pro-B ALL development highlights a so far unreported mechanism of functional Aid up-regulation in pro-B cells in the process of malignant transformation. While it was previously reported that physiologic Rag1 expression slows down leukemogenesis in p19Arf-deficient mice [30], our data extend these findings to Aid and suggest that both Rag1 and Aid act as negative regulators in pro-B cells.

In normal pro-B and pre-BI cells AID and RAG1 are repressed by IL-7R-signaling (active JAK-STAT/PI3K-AKT) [40–42], since both AID and RAG1 can induce negative selection and cell death at this early B-cell stage [8, 25]. Nevertheless, pro-B ALL tumors in AR mice displayed significant Aid upregulation. This detectable Aid expression and the fact that Aid deficiency lead to a dose-dependent pro-B ALL acceleration, suggests a mechanism that is similar to the clearance of autoreactive cells to establish central tolerance via Aid induced DNA damage and subsequent p53-mediated induction of cell death [26, 43]. In our model, we extend this role of Aid to arrested pre-leukemic pro-B cells which have not yet undergone Rag1 mediated V(D)J-recombination.

Although it was shown that the IL-7R safeguards pre-B cells against premature AID activation [8], pro-B cells oscillate between IL-7R high and low states [40]. Since the Rag1 negative pro-B cell arrested population in our model is highly susceptible to IL-7 exhaustion and subsequent cell death, disruption of IL-7R signaling could enable the Aid expression necessary to clear Rag1 deficient pre-leukemic clones, which would be a mechanistic explanation for the dose-dependent pro-B ALL acceleration in Aid deficient counterparts. The fact that low levels of pro-B ALL development can be observed in AR mice even in the presence of Aid can be accounted for by the acquisition of pro-survival mutations affecting the IL-7R pathway. To that effect, whole exome sequencing of AR, ARa and ARA pro-B ALLs identified highly recurrent somatic mutations in both *Dnm2* and *Jak3.* Loss of function mutations in Dnm2 have recently been linked to increased IL-7R density on the surface of murine Lmo2 transgenic T-ALLs [34]. By up-regulating IL-7R surface expression, arrested pro-B cells could respond to limited IL-7 availability, due to aberrant accumulation and increased consumption [44]. Additional mutations in Jak3 p.R653H and p.V670A, which confer constitutive active downstream signaling via pStat5 and arise shortly before blast propagation [33], render the cells IL-7 independent and counteract the negative regulation imposed by Aid.

The observation that B220^+^ sorted cells from healthy ARA mice showed increased proliferative potential, when compared to Aid competent AR mice, suggests that loss of Aid already affects certain intrinsic cell properties of IL-7 dependent pro-B cells. So far, increased proliferation of Aid-deficient cells has been shown to be significant in immature B-cells, which displayed an advantage through increased self-renewal capacity, seen by comparing Aicda^+/+^ against Aicda^-/-^ stem cell populations [21]. Therefore, it will be interesting to explain these *in vitro* results by elucidating the differences between healthy AR and ARA pro-B cells on a general gene expression level, or even on an epigenetic basis.

While it was previously shown that AID expression is regulated by PI3K [45], these findings were recently extended further by uncovering that PI3Kγδ or Bruton's kinase inhibitor treatment of mature B-cells leads to enhanced AID expression, which causes increased genomic instability [46]. Combining these insights with our own data highlights the importance of a tightly regulated Aid expression during the life of a B-cell. While loss of Aid in pro-B cells promoted pro-B ALL in our model and is known to impair central B-cell tolerance in immature B-cells [26], its upregulation contributes to the clonal evolution of leukemia in small pre-BII cells [8] and can induce genomic instability in mature B-cells [46].

In summary, we propose Aid as a negative regulator in Rag1 deficient pro-B cells, whereby Aid clears aberrant pro-B cells that are leukemia prone. Therefore, we extend the role of Aid in clearing autoreactive B-cells to establish B-cell tolerance, to the negative regulation of pro-B cells that are susceptible to malignant transformation in the specific context of Rag1 deficiency and pro-B ALL development.

## MATERIALS AND METHODS

### Generation of *p19Arf*^-/-^*/Rag1*^-/-^*/Aid*^-/-^ mice

The *p19Arf*^-/-^/*Rag1*^-/-^ mice were generously provided by Marina Cavazzana [30], Imagine, Paris and crossed back on Aid deficient mice [16] kindly provided by Tasuku Honjo, to obtain the mouse cohorts *p19Arf*^-/-^/*Rag1*^-/-^/*Aid*^-/-^ and *p19Arf*^-/-^/*Rag1*^-/-^/*Aid*^+/-^. Recipient C57BL/6 wildtype mice were obtained from Janvier Laboratories. Animals were housed in a specific, pathogen-free animal facility at the ZETT/Heinrich-Heine University Duesseldorf and experiments were performed in compliance with the German State Agency for Nature, Environment and Consumer Protection (LANUV).

### Cell culture

Iscove's modified Dulbecco's medium supplemented with 50 μM β-mercaptoethanol, 1 mM L-glutamine, 2% heat-inactivated fetal calf serum, 1mM penicillin-streptomycin (Life Technologies) and 0.03% (w/v) primatone RL (Sigma) was used for pro-B cell culture experiments. Pro-B cells isolated by MACS-sorting for B220^+^ (Miltenyi Biotec) from mouse BM were cultured on Mitomycin-C-treated ST2 feeder cells in IMDM medium containing IL-7 (5 ng/ml) (R&D Systems).

### Proliferation & BrdU assay

3×10^5^ healthy pro-B cells of *p19Arf*^-/-^*Rag1*^-/-^, *p19Arf*^-/-^*Rag1*^-/-^*Aid*^+/-^ and *p19Arf*^-/-^*Rag1*^-/-^*Aid*^-/-^ mice were seeded on day 1 in a 6-well plate. Cell numbers were determined every 24 hours using a Vi-Cell XR (Beckman Coulter). Cells from day 3 were further analyzed for proliferation with the FITC BrdU Flow Kit (BD Biosciences). The cells were pulse labeled with BrdU for 1 hour and analyzed on a FACSCalibur^TM^ Flow Cytometer according to the manufacturer's protocol (ARA *n* = 4, ARa *n* = 3, AR *n* = 3; biological replicates).

### Immunoblot analysis

Leukemic bone barrow cells from *p19Arf*^-/-^*Rag1*^-/-^, *p19Arf*^-/-^*Rag1*^-/-^*Aid*^+/-^ and *p19Arf*^-/-^*Rag1*^-/-^*Aid*^-/-^ mice, switch culture stimulated spleen cells from WT C57BL/6 mice (LPS (20 μg/ml) + IL4 (25 ng/ml) treatment for 4 days) or BaF3 cells (with and without expression of murine Jak3 V670A and after 4h of IL-3 depletion) were lysed in RIPA buffer (50 mM Tris pH 8.0, 150 mM NaCl, 0,5 % Sodiumdeoxycholate, 1 % NP-40 substitute, 0,1 % SDS) containing protease/phosphatase inhibitors (Roche Diagnostics). Immunoblotting was carried out using the following antibodies: anti-AID clone L7E7 1:1000 (Cell Signaling), anti-phospho-Stat5 D47E7 (Tyr694) 1:1000 (Cell Signaling), anti-Stat5 1:1000 (Cell Signaling) and anti-β-Actin clone AC-74 1:10000 (Sigma-Aldrich). Detection was carried out using anti-mouse horseradish peroxidase conjugates (Santa Cruz Biotechnology) with an ECL system (Thermo Scientific) (*n* = 3).

## SUPPLEMENTARY MATERIALS FIGURES AND TABLES






