# Supplementary material for: Activation-induced cytidine deaminase prevents pro-B cell acute lymphoblastic leukemia by functioning as a negative regulator in Rag1 deficient pro-B cells
Source: Oncotarget. 2017 Sep 7;8(44):75797–807. doi: 10.18632/oncotarget.20563 (PMC5652663; doi:10.18632/oncotarget.20563)
Supplement: Supplementary file 1 [file oncotarget-08-75797-s001.pdf]

Activation-induced cytidine deaminase prevents pro-B cell acute lymphoblastic leukemia by functioning as a negative regulator in Rag1 deficient pro-B cells

SUPPLEMENTARY MATERIALS

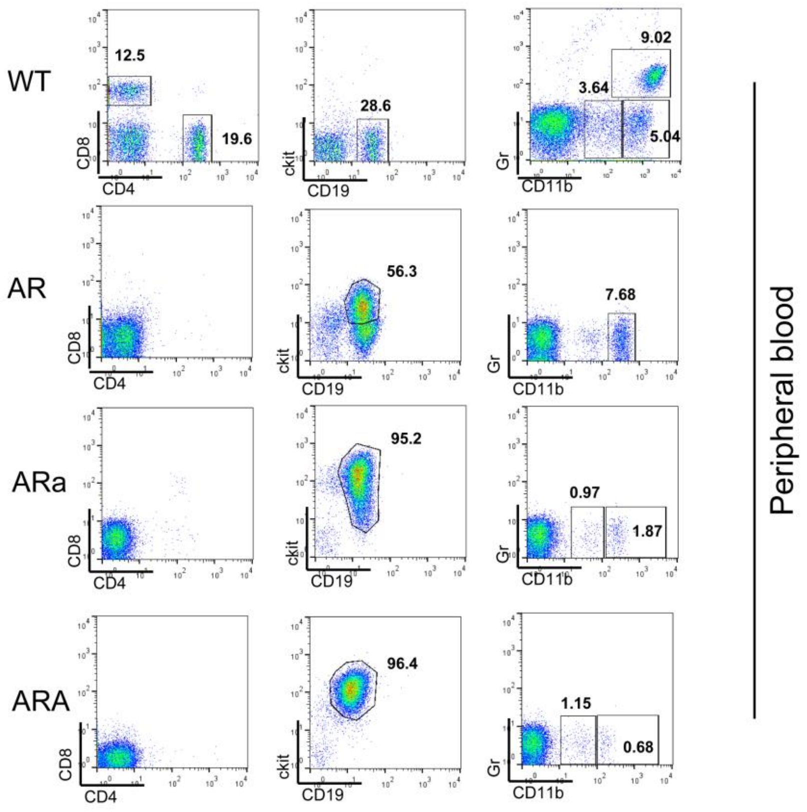

Supplementary Figure 1 (**Figure S1**): Flow cytometric analysis of peripheral blood of leukemic *p19Arf*$^{-/-}$*Rag1*$^{-/-}$ (AR), *p19Arf*$^{-/-}$*Rag1*$^{-/-}$*Aid*$^{+/-}$ (ARa) and *p19Arf*$^{-/-}$*Rag1*$^{-/-}$*Aid*$^{-/-}$ (ARA) mice. Representative plots of T-, B- and myeloid cell subsets are shown and compared to wildtype (WT) mice.

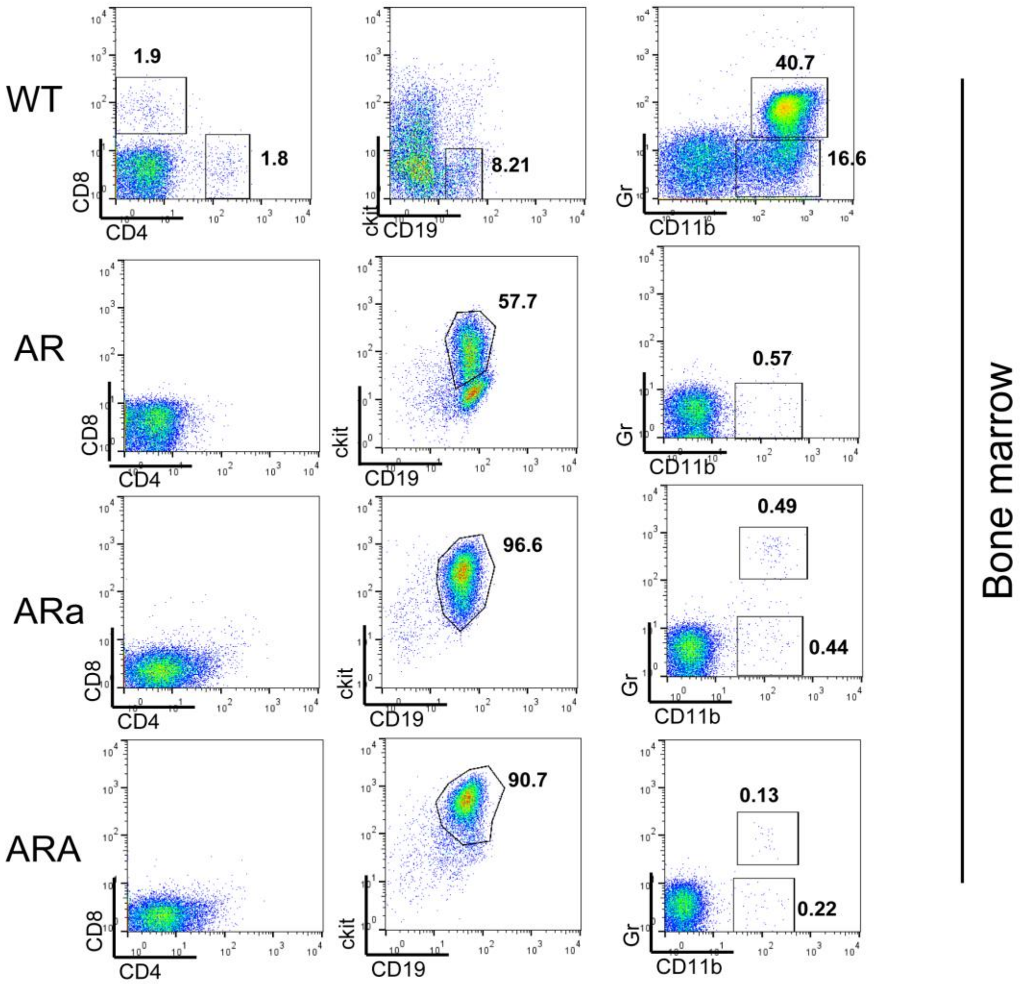

Supplementary Figure 2 (**Figure S2**): Flow cytometric analysis of bone marrow of leukemic $p19Arf^{-/-}Rag1^{-/-}$ (AR), $p19Arf^{-/-}Rag1^{-/-}Aid^{+/-}$ (ARa) and $p19Arf^{-/-}Rag1^{-/-}Aid^{-/-}$ (ARA) mice. Representative plots of T-, B- and myeloid cell subsets are shown and compared to wildtype (WT) mice.

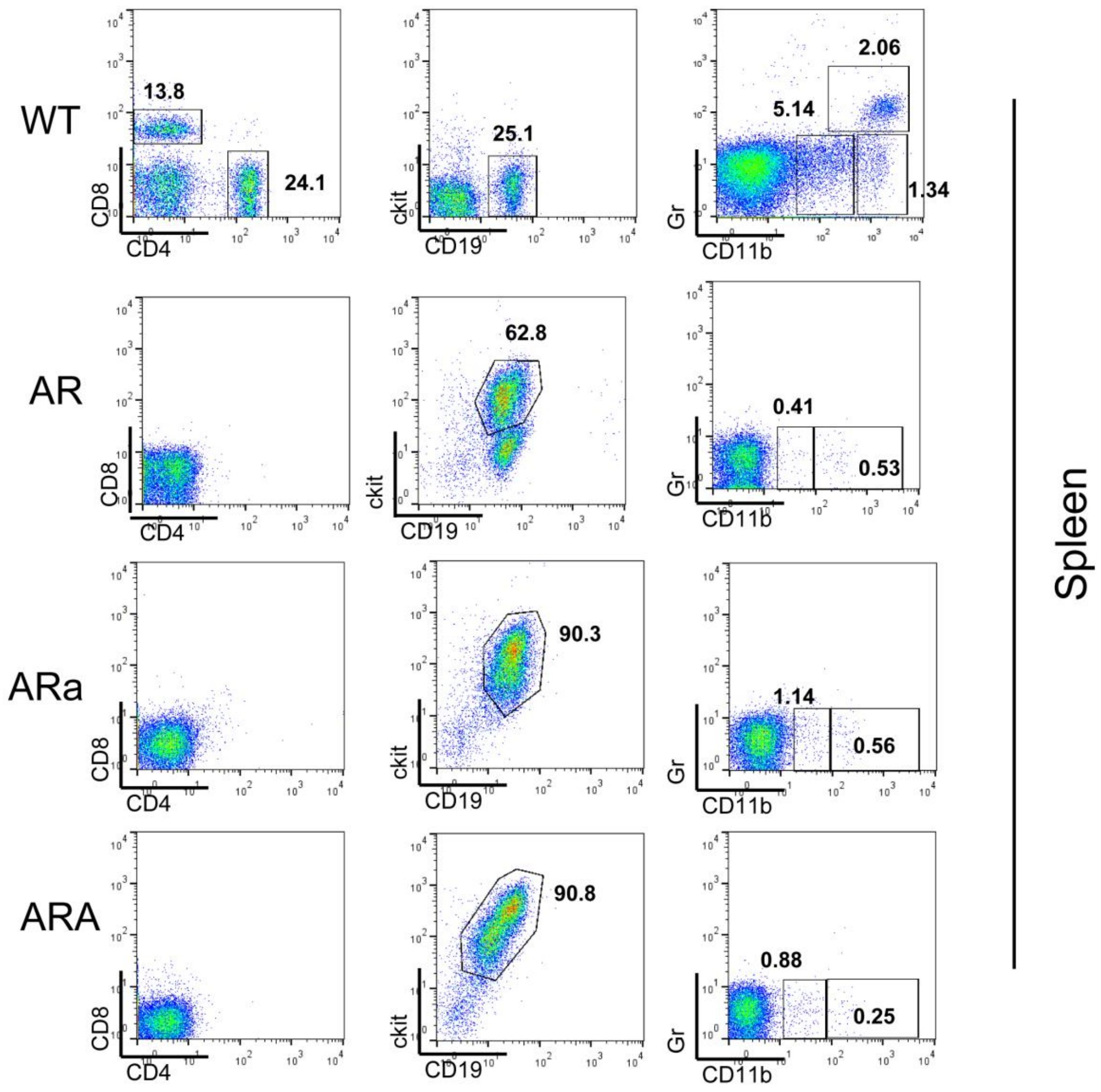

Supplementary Figure 3 (**Figure S3**): Flow cytometric analysis of spleen of leukemic *p19Arf*$^{-/-}$*Rag1*$^{-/-}$ (AR), *p19Arf*$^{-/-}$*Rag1*$^{-/-}$*Aid*$^{+/-}$ (ARa) and *p19Arf*$^{-/-}$*Rag1*$^{-/-}$*Aid*$^{-/-}$ (ARA) mice. Representative plots of T-, B- and myeloid cell subsets are shown and compared to wildtype (WT) mice.

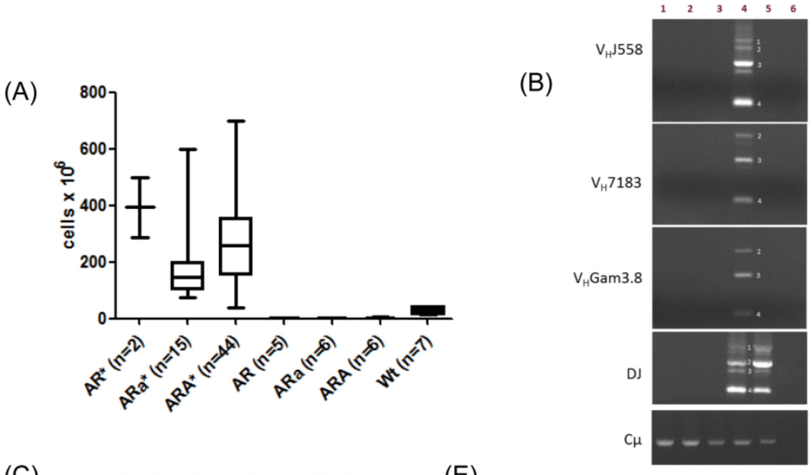

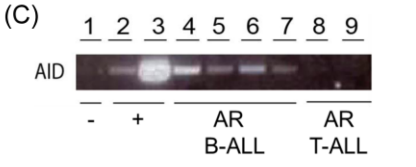

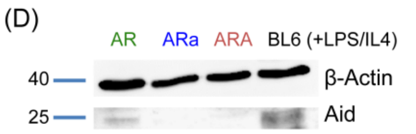

(E)

	Total cells x 10^5	Cells in S-Phase x 10^5	Average S-Phase x 10^5	StabW	Average % of cells in S-Phase
**AR**	29.8	8.3			
	24.3	6.1			
	23.1	6.3	6.90	1.2	26.8 ± 4.8
**ARa**	33.3	10.4			
	24.0	7.4			
	34.8	13.0	10.27	2.8	33.4 ± 9.2
**ARA**	49.2	23.5			
	34.8	12.5			
	41.7	14.7			
	40.5	13.2	15.98	5.1	38.5 ± 12.2

Supplementary Figure 4 (**Figure S4**): Extended information for murine pro-B leukemias.

(A) Number of cells in leukemic spleens from diseased (*) *p19Arf*$^{-/-}$*Rag1*$^{-/-}$ (AR), *p19Arf*$^{-/-}$*Rag1*$^{-/-}$*Aid*$^{+/-}$ (ARa) and *p19Arf*$^{-/-}$*Rag1*$^{-/-}$*Aid*$^{-/-}$ (ARA), as well as healthy AR, ARa and ARA mice, compared to C57BL/6J wildtype (Wt) mice.

(B) Immunoglobulin V(D)J-recombination in AR (lanes 1,2,3) pro-B ALLs. Heavy-chain gene rearrangements in infiltrated tissues of diseased mice were analyzed by PCR. Sorted CD19$^+$ B-cells (lane 4) from the spleens of healthy C57BL/6 mice serve as a control for polyclonal BCR rearrangements, while thymocytes (lane 6) were included as a negative control. Infiltrated tissues show only the cμ heavy chain.

(C) Aid expression visualized on an agarose gel shows B-cell specific Aid upregulation in *p19Arf*$^{-/-}$*Rag1*$^{-/-}$ (AR) pro-B ALLs (lanes 4-7), while no Aid transcripts were detected in mice from the same background that developed T-ALLs (lanes 8-9). LPS and IL-4 stimulated mature splenic murine B-cells served as positive (+) (lanes 2-3) and distilled water as negative (-) control (lane 1).

(D) Replicate of western blot showing the presence of Aid protein in *p19Arf*$^{-/-}$*Rag1*$^{-/-}$ (AR) pro-B ALLs. LPS (20 µg/ml) and IL-4 (25 ng/µl) stimulated B220^{+} splenic B-cells from wildtype mice served as positive control. Beta-Actin was used as loading control.

(E) Number of *in vitro* cultured total cells and cells in S-phase of healthy *p19Arf*$^{-/-}$*Rag1*$^{-/-}$ (AR), *p19Arf*$^{-/-}$*Rag1*$^{-/-}$*Aid*$^{+/-}$ (ARa) and *p19Arf*$^{-/-}$*Rag1*$^{-/-}$*Aid*$^{-/-}$ (ARA) mice counted for the BrdU assay described in **Figure 4E**.

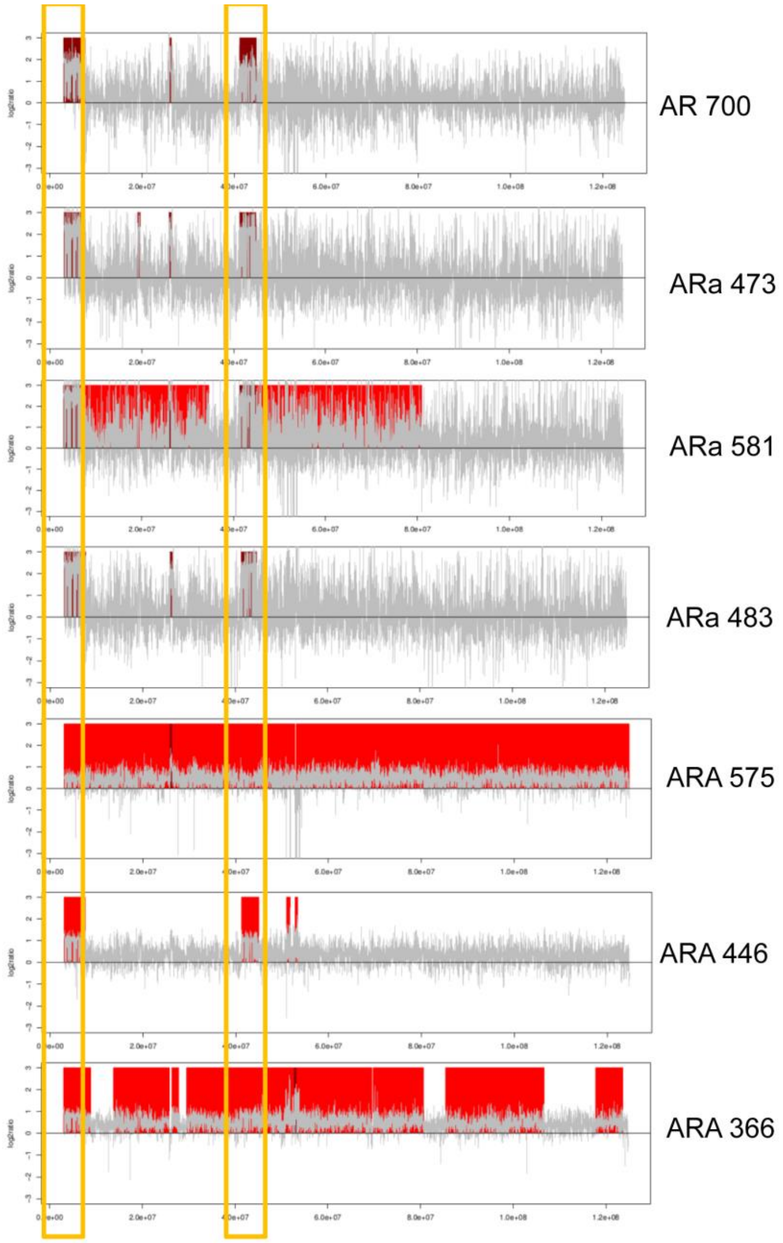

Supplementary Figure 5 (**Figure S5**): Copy number variation analysis using EXCAVATOR2 showing Chr. 14 gains (red) of all AR, ARa and ARA pro-B ALLs

analyzed. Orange boxes indicate recurrently amplified regions in all samples (region: 3010202 – 7542944 and region: 41183354 – 44706288).

Supplementary Table 1 (**Table S1**): Differentially expressed genes in bone marrow tumors from AR mice compared to tumors from ARA mice.

Supplementary Table 2 (**Table S2**): Genes on Chr. 14 recurrently amplified in all ARA, AR and AR pro-B ALLs analyzed.

Supplementary Methods:

Animals

The *p19Arf*$^{-/-}$/*Rag1*$^{-/-}$ mice were generously provided by Marina Cavazzana [1], Imagine, Paris and crossed back on Aid deficient mice [2] kindly provided by Tasuku Honjo, to obtain *p19Arf*$^{-/-}$/*Rag1*$^{-/-}$/*Aid*$^{-/-}$ mice. Recipient wildtype mice were obtained from Janvier Laboratories. Animals were housed in a specific, pathogen-free animal facility at the ZETT/Heinrich-Heine University Duesseldorf and experiments were performed in compliance with the LANUV. Only female mice were included in the study with study duration of up to 52 weeks. Upon signs of disease, mice were sacrificed and subjected to standard necropsy procedures. All major organs were examined under the dissecting microscope. Tissue samples were taken from homogenous portions of the resected organ and fixed immediately after excision. Differences in Kaplan-Meier survival plots of transgenic and wildtype mice were analyzed using the log-rank (Mantel-Cox) test.

FACS analysis

Nucleated cells were obtained from total mouse bone marrow (flushing from the long bones), peripheral blood, lymph nodes or spleen. Red blood cell lysis was performed with NH_4Cl. The samples and the data were acquired using a FACSCalibur™ (BD Biosciences) Flow Cytometer and analyzed using Flowjo software. Specific fluorescence of FITC, PE, PerCP and APC excited at 488 nm (0.4 W) and 633 nm (30 mW), respectively, as well as known forward and orthogonal light scattering properties of mouse cells were used to establish gates. For each analysis, a total of at least 20000 cells were assessed (ARA n=45, ARa n=18).

Antibodies were all purchased from BD Biosciences. The following antibodies were used for flow cytometry: CD8 FITC (53-5.8, 1:20), CD4 APC (RM4-5, 1:30), CD19 FITC (1D3, 1:20), CD117/c-kit PE (2B8, 1:20), IgM APC (II/41, 1:20), CD11B/Mac1 FITC (M1/70, 1:10), Ly-6G/Gr1 (RB6-8C5, 1:40).

Histology

Animals were sacrificed by cervical dislocation. Spleen tissue samples from *p19Arf*$^{-/-}$/*Rag1*$^{-/-}$/*Aid*$^{-/-}$ mice, which developed pro-B ALL and a WT control mouse were harvested, fixed in formalin, embedded in paraffin, and cut into 2-μm-thick slices. Slides were conventionally stained with hematoxylin-eosin reagent (n=3).

V(D)J recombination assay

Immunoglobulin rearrangements were amplified by PCR using the primers below. Cycling conditions consisted of an initial heat-activation at 95ºC followed by 31-37 cycles of denaturation for 1 min at 95ºC, annealing for 1 min at 65ºC, and elongation for 1 min 45 s at 72ºC. This was followed by a final elongation for 10 min at 72ºC. To determine the DNA sequences of individual V(D)J rearrangements, the PCR fragments were isolated from the agarose gel and cloned into the pGEM-Teasy vector (Promega); the DNA inserts of at least ten clones corresponding to the same PCR fragment were then sequenced (ARA n=2, ARa n=2, AR n=3). The following primer pairs were used:

V_HJ558	forward	CGAGCTCTCCARCACAGCCTWCATGCARCTCARC
	reverse	GTCTAGATTCTCACAAGAGTCCGATAGACCCTGG
V_H7183	forward	CGGTACCAAGAASAMCCTGTWCCTGCAAATGASC
	reverse	GTCTAGATTCTCACAAGAGTCCGATAGACCCTGG
V_HQ52	forward	CGGTACCAGACTGARCATCASCAAGGACAAYTCC
	reverse	GTCTAGATTCTCACAAGAGTCCGATAGACCCTGG
DH	forward	TTCAAAGCACAATGCCTGGCT
	reverse	GTCTAGATTCTCACAAGAGTCCGATAGACCCTGG
Cμ	forward	TGGCCATGGGCTGCCTAGCCCGGGACTT
	reverse	GCCTGACTGAGCTCACACAAGGAGGA

Mouse exome library preparation and next generation sequencing

Sample acquisition: The AllPrep DNA/RNA Mini Kit (Qiagen) was used to purify DNA according to the manufacturer’s instructions.

Exome library preparation and next generation sequencing: Exome library preparation was performed using the Agilent SureSelectXT Mouse All Exon kit with modifications. Targeted capture by hybridization to an RNA library was performed according to the manufacturer’s protocol. Purification and enrichment of the captured library was achieved by binding to MyOne Streptavidin T1 Dynabeads (LifeTechnolgies) and off-bead PCR amplification in the linear range. 2x100 bp sequencing with a 6 bp index read was performed using the TruSeq SBS Kit v3 on the HiSeq 2500 (Illumina) (ARA Germline/Tumor pairs n=3, ARa tumor n=3, AR tumor n=1).

Data analysis: Fastq files were generated by using BcltoFastq 1.8.4 (Illumina). BWA version 0.7.4. was used to align sequence data to the mouse reference genome (GRCm38.71). Conversion steps were carried out using Samtools followed by removal of duplicate reads (http://broadinstittute.github.io/picard). Local realignment around indels, SNP-calling, annotation and recalibration was facilitated by GATK 2.4.9. Mouse

dbSNP138 and dbSNP for the used mouse strains were used as training datasets for recalibration. Resulting variation calls were annotated by Variant Effect Predictor using the Ensembl database (v70) and imported into an in-house MySQL database to facilitate automatic and manual annotation, reconciliation and data analysis by complex database queries. Loss of function prediction scores for PolyPhen2 and SIFT were extracted from this Ensemble release.

Somatic calls were produced using MuTect [3]. Further filtering was applied to consider only entries with at least 9% difference in allele frequency, between tumor and normal samples, for further analysis. Cancer related genes were determined by translating the cancer gene consensus from COSMIC using ENSEMBL's biomart. For the detection of copy number alterations, CNV profiles were calculated with an adapted version of EXCAVATOR2 [4]. Hard-coded human chromosomes in the source code were adapted to accomodate a mouse genome. The required mappability index for 100bp reads was calculated using the gem library (http://algorithms.cnag.cat/wiki/The_GEM_library), conversion was done with BigWig tools from UCSC [5]. In addition to the animals shown, 15 mice with a non-related genetical background were used to generate a normalization control.

Sequencing

Mutations were validated using Sanger sequencing on a 3130 Genetic Analyzer (Applied Biosystems). The following primer pairs were used (5' → 3'): mJak3_Exon15 *forward*: CGGGATGTGGGGCTTTAACT, *reverse:* GCAGACACGGGGTATAGTGG; mDnm2_Ex8 *forward*: GCCGTGCAGAAAACTCTGTC, *reverse*: GGCCAAGACAAA GACCAAGC; mDnm2_Ex9 *forward*: TAGCCACTGTTCCTGCTGTG, *reverse*: CAGCCAAAACACACGCACTT (ARA n=14, ARa n=13 for Jak3 and n=14 for Dnm2, AR n=4).

Quantitative Real Time PCR

RNA was prepared using the AllPrep DNA/RNA Mini Kit (Qiagen). 1 µg of RNA was reverse transcribed into cDNA with the QuantiTect Reverse Transcription Kit according to manufacturer's instructions (Qiagen). Aid mRNA levels were determined using the TaqMan gene expression assay for mouse *Aicda* Mm00507774_m1 (Applied Biosystems) and were normalized to the corresponding GAPDH levels (Mm03302249_g1 – Applied Biosystems). In short, 40 ng/µl cDNA per sample were run in triplicate with the following program on a CFXConnect^{TM} Real time system (BioRad): 95°C 10 min, 95°C 15 sec, 60°C 1 min. Denaturation and annealing/extension were performed for 45 cycles (n=3).

Microarrays:

All samples where processed together on one Agilent Mouse Microarray (GPL13912-20417) chip. All arrays where individually loaded and filtered according to standard quality control practices (ControlType=0,gIsPosAndSignif=1,gIsWellAboveBG=1). After that, all arrays were quantile normalized together. T-tests were used to compare both groups and corrected by using the Bonferroni-Holm correction for multiple comparisons. The following open source python libraries were used: pandas (0.19.0), scipy (0.18.1), statsmodels (0.6.1), scikit-learn (0.18), matplotlib (1.5.1), seaborn (0.7.1) and R (3.3.2). ARA n=4, AR n=2.

Bone marrow transplantation

5 x 10^6 leukemic bone marrow cells of *p19Arf*$^{-/-}$*Rag1*$^{-/-}$*Aid*$^{-/-}$ mice were injected into non-irradiated recipient wild-type mice (C57BL/6). After first signs of illness, the mice were sacrificed and assessed for pro-B ALL development by FACS analysis (n=2).

Statistical analysis

All statistical analyses were performed using the GraphPad Prism software (GraphPad Software Inc). Survival was analyzed according to the Kaplan-Meier method and survival curves were compared in a log-rank test.

Supplemental References:

1. Hauer J, Mullighan C, Morillon E, Wang G, Bruneau J, Brousse N, Lelorc'h M, Romana S, Boudil A, Tiedau D, Kracker S, Bushmann FD, Borkhardt A, et al. Loss of p19Arf in a Rag1(-/-) B-cell precursor population initiates acute B-lymphoblastic leukemia. Blood. 2011; 118: 544-53. doi: 10.1182/blood-2010-09-305383.
2. Muramatsu M, Kinoshita K, Fagarasan S, Yamada S, Shinkai Y, Honjo T. Class switch recombination and hypermutation require activation-induced cytidine deaminase (AID), a potential RNA editing enzyme. Cell. 2000; 102: 553-63. doi:
3. Cibulskis K, Lawrence MS, Carter SL, Sivachenko A, Jaffe D, Sougnez C, Gabriel S, Meyerson M, Lander ES, Getz G. Sensitive detection of somatic point mutations in impure and heterogeneous cancer samples. Nat Biotechnol. 2013; 31: 213-9. doi: 10.1038/nbt.2514.
4. D'Aurizio R, Pippucci T, Tattini L, Giusti B, Pellegrini M, Magi A. Enhanced copy number variants detection from whole-exome sequencing data using EXCAVATOR2. Nucleic Acids Res. 2016; 44: e154. doi: 10.1093/nar/gkw695.
5. Kent WJ, Zweig AS, Barber G, Hinrichs AS, Karolchik D. BigWig and BigBed: enabling browsing of large distributed datasets. Bioinformatics. 2010; 26: 2204-7. doi: 10.1093/bioinformatics/btq351.
